# Protein Aggregation in *E. coli* : Short Term and Long Term Effects of Nutrient Density

**DOI:** 10.1371/journal.pone.0107445

**Published:** 2014-09-11

**Authors:** Ulfat I. Baig, Bharati J. Bhadbhade, Dincy Mariyam, Milind G. Watve

**Affiliations:** 1 Department of Microbiology, Abasaheb Garware College, Pune, Maharashtra, India; 2 Department of Biology, Indian Institute of Science Education and Research, Pune, Maharashtra, India; CSIR-Central Drug Research Institute, India

## Abstract

During exponential growth some cells of *E. coli* undergo senescence mediated by asymmetric segregation of damaged components, particularly protein aggregates. We showed previously that functional cell division asymmetry in *E. coli* was responsive to the nutritional environment. Short term exposure as well as long term selection in low calorie environments led to greater cell division symmetry and decreased frequency of senescent cells as compared to high calorie environments. We show here that long term selection in low nutrient environment decreased protein aggregation as revealed by fluorescence microscopy and proportion of insoluble proteins. Across selection lines protein aggregation was correlated significantly positively with the RNA content, presumably indicating metabolic rate. This suggests that the effects of caloric restriction on cell division symmetry and aging in *E. coli* may work via altered protein handling mechanisms. The demonstrable effects of long term selection on protein aggregation suggest that protein aggregation is an evolvable phenomenon rather than being a passive inevitable process. The aggregated proteins progressively disappeared on facing starvation indicating degradation and recycling demonstrating that protein aggregation is a reversible process in *E. coli*.

## Introduction

Senescence in bacteria in spite of conditions favoring growth became a focus of active interest only recently. In *Caulobacter* [1], [2] there is a clear distinction between mother cell and daughter cell and the mother cell has been shown to slow down and stop reproducing after a certain number of division cycles. The division in such a species is morphologically and functionally asymmetric. For some time it was thought that there is no senescence or natural death in bacteria that undergo a morphologically symmetric division. On this background the demonstration by Stewart *et al* [3] that even in *E. coli* the cell division was functionally asymmetric and that the “old pole” cell gradually slowed down its growth and stopped dividing came as a surprise. This finding triggered both theoretical and empirical work and gave rise to many new possibilities.

Aging in *E. coli* is thought to happen because of asymmetric segregation of damaged components to the daughter cells. The damaged components were later shown to be mainly, if not exclusively, protein aggregates (PAs) [4], [5], [6]. There appear to be two possible alternative evolutionary interpretations of the asymmetric segregation phenomenon. According to one senescence is inevitable and cellular polarization is suggested to have evolved to restrict senescence to one daughter lineage [7], [8]. According to the other, there are two possible ways of handling damaged proteins. One is to repair or recycle the damaged components and the other is to dump them asymmetrically in one cell to leave the other free of damage. Mathematical models show that repair or recycle could be a better strategy in severely nutrient limited environments and asymmetric dumping may work better in rich nutrient environments [9]. These models expect that the extent of protein aggregation and/or the asymmetry of segregation would be responsive to different nutritional environments.

In the initial concept of bacterial aging the distinction of old pole and new pole was thought to be critical, with the old pole cell always retaining the aggregate and senescing [3], [4], [8]. However there is a possibility that there can be pole flipping, i. e. the damaged components may drift away from the morphologically old pole with some probability. Even if the probability is small the old pole cell may rejuvenate and maintain its growth rate over a long time [10]. Some studies have indicated that the old pole cell is not always the slower of the two in terms of growth and next division [11]. Therefore the role of morphologically old and new poles may be less important than assumed earlier, nevertheless the importance of asymmetric segregation of protein aggregates remains more robust theoretically as well as empirically.

Protein aggregation is associated with aging and related disorders across wide diversity of taxa including humans [12], [13], [14]. Because all organisms face the important challenges of protein misfolding and aggregation, strategies to cope with the problem are expected to have evolved and be conserved [15], [16]. However our understanding of the evolution of protein handling mechanisms is as yet very poor. It is interesting to ask why components of proteostasis machinery are not constitutively expressed at high levels, or why it did not evolve to perfection; whether an efficient proteostasis is impossible or whether the cost benefits of a highly efficient system are not favorable. This question cannot be context independent and it is likely that the cost-benefits of efficient protein handling systems are more beneficial under certain conditions and costly under others. One of the suggested costs in multicellular organisms is the increased potentiation of cancerous growth [17]. Alternatively a trade-off between somatic maintenance and reproduction may decide an optimum level of investment in proteostasis mechanisms in somatic cells [18]. This optimum could be much short of perfection and therefore protein aggregation is commonly observed. Understanding protein folding and mechanisms of PA formation in bacteria can serve as a simpler model for the mechanics as well as the evolutionary dynamics of proteostasis mechanisms which can ultimately help us understand how and why human conformational diseases progress.

In bacteria aggregate formation and features are influenced by various growth conditions such as temperature, pH [19], culture phase [5] and glucose/oxygen availability [20]. All proteins in inclusion bodies were initially considered as misfolded, but it has been shown that some active polypeptides are present inside aggregates [21] presumably due to accidental trapping. Misfolded proteins are presumably deposited in the nucleoid free space because of molecular crowding and these aggregates get deposited more frequently at poles [4], [6]. This leads to asymmetric inheritance of denatured proteins which are a burden for old pole cell. Cells that are less burdened with aggregates divide faster as compared to the ones with a greater burden [16].

Since the association of protein aggregates with asymmetric damage segregation, asymmetry in cell growth and division time and aging has been shown by many studies, we take these associations for granted and investigate,

- Whether protein aggregation is an inevitable phenomenon or the cell has some control over it. Is it possible to avoid or minimize protein aggregation and whether the cell executes these mechanisms all the time or strategically under certain conditions?
- Whether asymmetric segregation of protein aggregation is an inevitable phenomenon or is a strategic behaviour subject to differential natural selection under different environments?
- Whether protein aggregates are outright waste products or whether they have any beneficial role under some conditions?

A common approach to study protein aggregation in the context of aging is to follow individual cells and their subsequent daughters for a few generations [3], [4], [6], [10]. This is done using fluorescence microscopy to reveal the protein aggregates. A possible objection to this approach is that the cells under observation are continuously or repeatedly exposed to UV light during imaging which might alter the fate of proteins [22], [23]. Since the extent of alteration in protein handling during fluorescence microscopy has not been estimated we use a cross sectional approach where cells are not observed longitudinally but a growing population is sampled at a cross section in time. This does not reveal information of old or new poles and mother or daughter cells but inferences based on the statistical distribution of aggregates are nevertheless possible. The cross sectional data can therefore be cleaner and important despite the limitation that the inferences are necessarily statistical and indirect.

## Materials and Methods

Strains used: We studied MGAY, obtained from A. Lindner (INSERM, France) which constitutively expresses yellow fluorescent protein (YFP) fused to the C terminus of Inclusion body protein (Ibp) A under the control of the endogenous chromosomal IbpA promoter. This allows visualization of protein aggregates in the cells under fluorescence microscopy [4] [24].

### Selection regime

Similar to Lele *et al* [11] we grew the above strain continuously under two concentrations of glucose in a mineral medium. On agar media with high (10 mg/ml) or low (0.1 mg/ml) concentrations of glucose as the sole source of carbon and energy, cultures were serially transferred up to an estimated 1800 generations so that at the end of the prolonged sub-culturing we had three cell lineages, one each selected for either of the nutritional environments and one wild type. We denote the strain selected under high caloric environment as *H* and the one selected under low caloric environment as *L*. The current medium condition for the experiment is denoted by small letters *h* and *l* for high and low caloric conditions respectively (e.g. a lineage selected under high concentration but currently being grown in low concentration is denoted as *Hl*).

In addition we also used the lines selected by Lele *et al* [11] originating from *E. coli* KL16 and 2563 with an identical protocol for an estimated 3000 generations. These lines did not have the fluorescent tag and therefore could not be used for microscopic observation of protein aggregates but were subject to differential estimation of soluble and insoluble protein contents (see below) which is thought to be a surrogate of protein aggregation. Lele *et al* [11] used three ancestral strains of *E. coli,* but since some of the selected lines from one of them lost viability only two of the three were used for this study.

### Growth conditions

All the selection lines were maintained on agar media with the respective nutrient concentration (*h* or *l*). Before preparing for microscopy or cell composition studies they were grown in respective liquid media on shake flask at 180 rpm for 16–18 hours at 37°C.

The starvation experiment was performed with two alternative starvation conditions. In one the cells were continued to be incubated in the same medium after entering the stationary phase and were observed with fluorescence microscopy at every 12 hours up to 48 hours. Alternatively the cells in early stationary phase were harvested, washed in distilled water three times and then suspended in distilled water again and incubated at 37°C. Observations with fluorescence microscopy were made after every 12 hours up to 48 hours.

### Fluorescence microscopy

For the selection lines derived from *E. coli* MGAY, when the cells reached an absorbance 0.2 (600 nm), they were placed on a slide that was layered with a 1.5% agarose pad and covered with a cover slip to immobilize the cells. Observations were made using differential interference contrast (DIC) and fluorescence imaging on Zeiss Axioimager M-1 upright microscope (100X/1.40 Oil DIC M27) with a color digital camera controlled by Axio software 4.8. Images both DIC/fluorescence were taken using exposure time of 30 millisecond illumination. The resulting images were processed and analyzed using Image J 1.44c software [25]. The fluorescence image alone was used for quantification of fluorescence intensity reflecting aggregate size whereas composite image formed by merging DIC and fluorescence was used to determine the location of protein aggregates in a cell.

### Fluorescence intensity classification

Fluorescence given by the protein aggregates was quantified in arbitrary units using integrated fluorescence intensity corrected for the background. The aggregates were classified into four intensity classes namely 0 (cells with no visible aggregates), 1 with intensity <1500 units, 2 with intensities between 1500 and 3000 and 3 with intensities greater than 3000 units. In an experiment pertaining to the effect of starvation on PAs, classes were resolved at finer intervals with a larger sample size (500 to 800 cells per observation).

To characterize the position of protein aggregates in *E. coli* cells, we measured the distances of an aggregate from the nearest pole. The distance was normalized by the cell length. As a result the normalized positions range from 0 to 0.5 which were divided in to 5 classes. The class 0 to 0.1 represents the polar location and 0.4 to 0.5 represents central position.

### Estimation of RNA and protein content of cells

The RNA and soluble and insoluble protein concentrations of cells were determined. A density of 10^7^–10^8^ cells/ml was used for the studies in which RNA extraction was done by hot phenol method [25], [26]. RNA quality was further checked by Agilent 2100 bioanalyzer and the RIN number for the same was 8 or above. Estimation of nucleic acids was done by Thermo scientific Nano drop 2000 [27]. For the quantification of soluble and insoluble proteins exponentially growing cells were harvested from 10-liter cultures and then soluble and insoluble fractions were extracted by Maisonneuve *et al* method [5] and estimated by bichinconic acid method [28].

## Results

### Low nutrient concentration reduces protein aggregation

**Figure 1:**
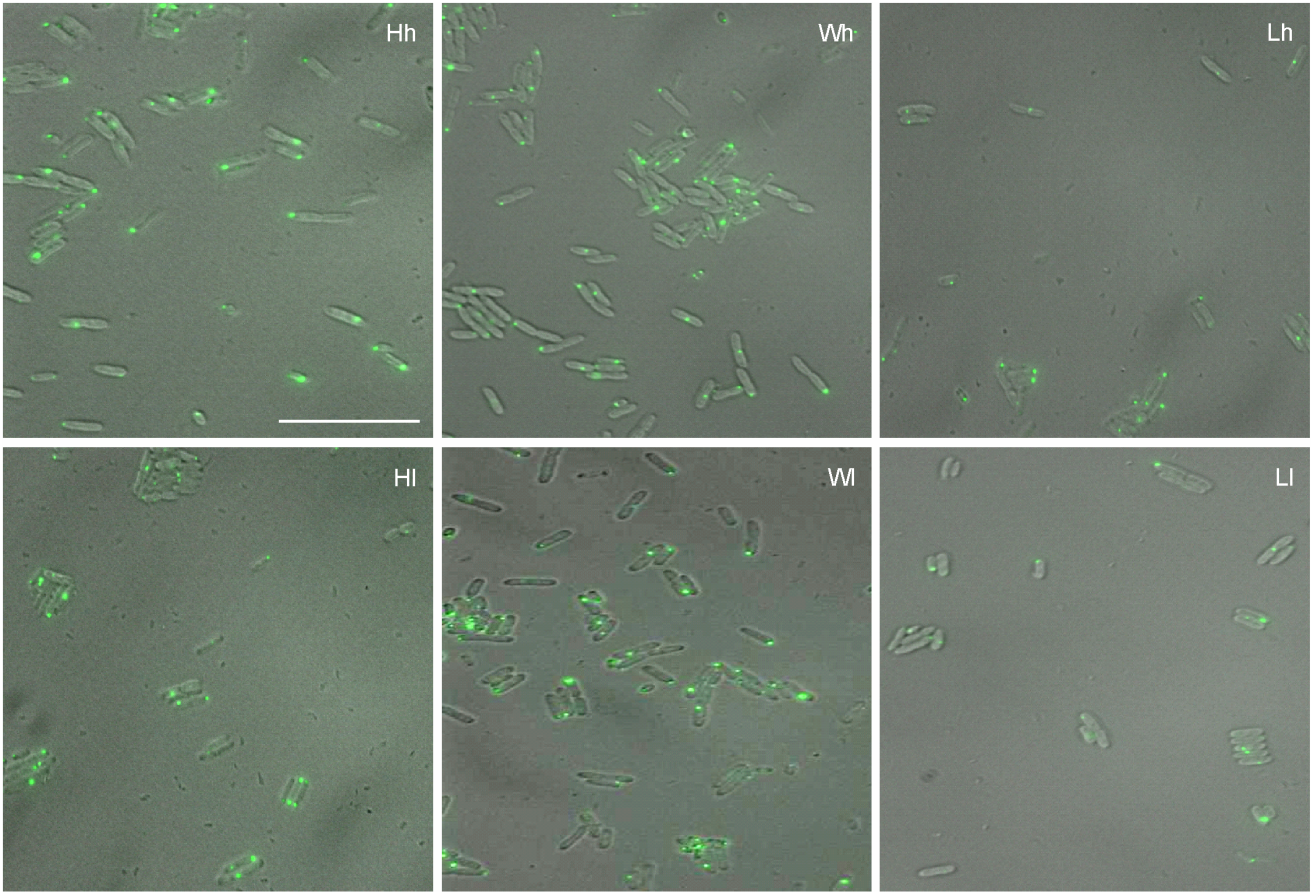
Superimposed DIC and fluorescence images of the three selection lines generated from MGAY in two current nutrient conditions. *W* stands for the wild type or ancestral strain. Selection was carried out for an estimated 1800 generation under constant low (*L*) and high (*H*) nutrient environments. Before microscopy all the three lines were grown in high (*h*) or low (*l*) glucose concentration for 16 hours. (e.g. *Hl:* a lineage selected under high nutrient concentration but currently being grown in low concentration). The same notation is used in all figures. All lineages showed visible protein aggregates under stress free conditions but the proportion of cells with aggregates and mean intensities differed across selection and current nutrient conditions. The scale bar represents 10 µ.

**Figure 2:**
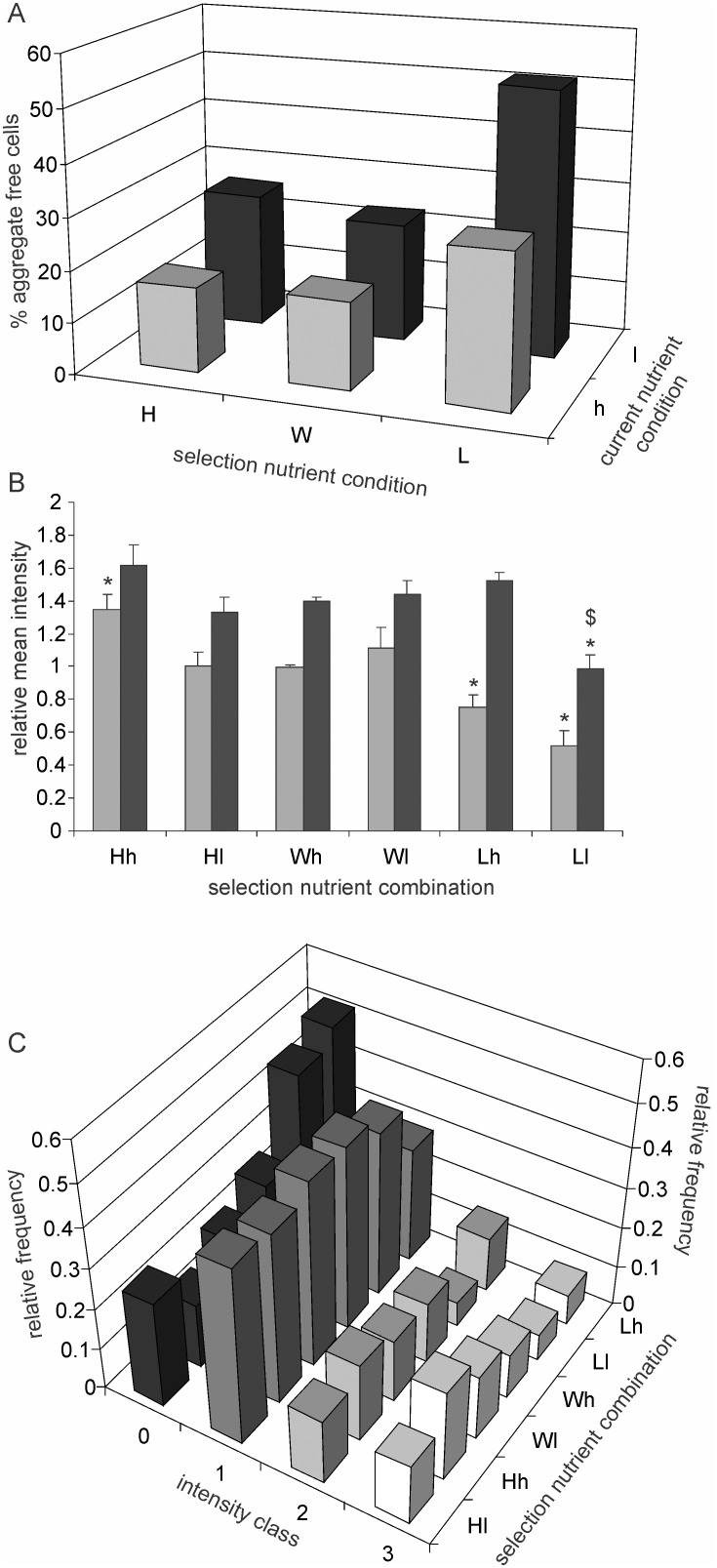
Effect of selection and current nutrient concentration on protein aggregation. (n = 200±20 each for data in Fig. 2A to C). A: The proportion of aggregate free cells: Both short term exposure and long term selection in low nutrient concentrations increased the proportion of aggregate free cells. (chi sq = 38.15, p<0.0001). B: The mean fluorescence intensities of cells (expressed relative to mean *Wh* including aggregate free cells) under the 6 selection nutrition combinations: The grey columns denote the average including cells without visible aggregates. The black columns denote average calculated by excluding the aggregate free cells. *denotes significant difference from Wh. $ denotes significant difference between corresponding *h* and *l* conditions (at p<0.05). C: Frequency distribution of cells with fluorescence intensity classes of aggregates: The modal class is 1 except in *Lh* and *Ll* where the modal class is 0. The proportion of cells in different intensity classes across the three selection lines differed significantly at high nutrient concentration (for *Wh, Hh* and *Lh* Chi square = 57.157, df = 6, P<0.0001) as well as low nutrient concentration (For *Wl*, *Hl* and *Ll* Chi square = 35.317, df = 6, P<0.0001). The effect of current nutrient concentration was not significant for *W* (Chi square = 2.35, df = 3, P = 0.503) but was significant for *H* (Chi square = 7.138, df = 3, P = 0.0676) and *L* (Chi square = 9.415, df = 3, P = 0.0243).

In selection lines derived from MGAY strain fluorescent protein aggregates were visible and quantifiable in all cell lineages under both *h* and *l* conditions in the absence of heat or any other external stress (Fig. 1). The proportion of aggregate free cells showed a gradient along both current nutrient concentration and selection axes. The maximum proportion of aggregate free cells was found in *Ll* whereas the minimum was in *Hh* (Fig. 2A). The mean fluorescence intensities of the aggregates calculated including zero intensity cells (aggregate free cells) showed a similar trend where both the effects of current concentrations and selection could be seen (Fig. 2B). After removing zero intensity cells from the average calculation both the effects remained in the same direction but were weaker (Fig. 2B). When the aggregates were categorized in 4 intensity classes, the frequency distribution was positively skewed in all the lines at both nutrient concentrations with the modal frequency being at zero for *Lh* and *Ll* and at intensity class 1 for all others (Fig. 2C). The decreasing frequency of larger aggregates is expected by the dynamics of cell division and asymmetric segregation. This distribution is similar to the one expected by a Leslie matrix model of stable age structure [9]. Since in an exponentially growing population with stable age class distribution the largest frequency is that of the youngest class, maximum frequency would be in the aggregate free or smaller aggregate class. The frequency of zero aggregate class will be determined by the relative rates of cell growth and division versus protein aggregation. It appears from the frequencies (>50%) that in *W* and *H* lineages under both *h* and *l* conditions, a visible aggregate appears in a cell in the first generation itself whereas in *Lh* and *Ll* since the frequency of cells showing aggregates is less than 50%, the first generation cells are presumably all free of visible aggregates.

Separating the effects of current nutrient concentration and selection, we compared the selection lines at a given nutrient concentration and nutrient concentrations for a given selection. It can be seen (Fig. 2C) that both current nutrient concentration and long term selection altered the distribution of intensity classes significantly. The main difference was in the zero intensity class, i.e. aggregate free cells. Among the cells with aggregates the differences between selection and current nutrition conditions were marginal. This indicates that the main difference caused by nutrient concentration and selection is likely to be in the rate of formation or early development of the aggregates and the difference in the later development could be smaller or absent.

**Figure 3:**
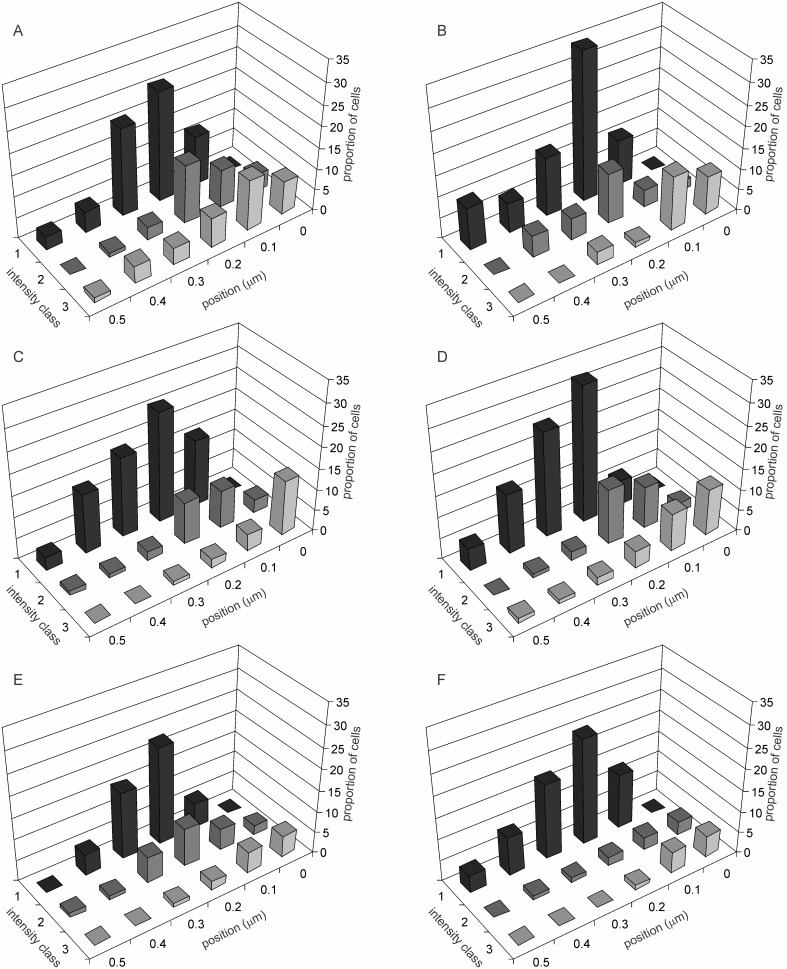
Spatial distribution of intensity classes under the six selection nutrition combinations. Only cells with visible aggregates are included. Positions 0 to 0.5 indicate the position of the aggregate along the cell length, 0 representing the nearest pole and 0.5 representing the centre of the cell. Note that the pattern is similar in all the lines. Aggregates in class 1 can be observed anywhere along the length with a subpolar peak. Older aggregates are more frequently polar. Similarity across selection demonstrates that although there is a difference in the rate of formation of the aggregates, once formed their further fate and spatial dynamics may be unaffected by selection or standing nutrient concentration. A–F represents *Hh, Hl, Wh, Wl, Lh* and *Ll* respectively.

Apart from the rates of aggregate formation and growth, the fate and spatial dynamics of the aggregates does not appear to be affected by the different selection and current conditions. This is apparent in the spatial distribution of the intensity classes in cells having aggregates (Fig. 3). The patterns in this distribution are remarkably similar across groups with the possible exception of *Ll* which has very few aggregates in class 2 and 3. Because of their smaller number it is difficult to decide whether their spatial distribution is significantly different from other lineages. The picture generalizable across selection and treatments is that smaller aggregates may originate anywhere in the cell with a higher probability at sub central or sub polar region. Larger aggregates tend to be polar more frequently, but they can be seen at other positions occasionally.

With a low frequency, cells with more than one aggregate at a time could be observed. Since there is only one aggregate in majority of the cells, it is obvious that this aggregate will go to only one of the daughter cells so that the distribution of aggregated proteins will be asymmetric. Since theoretical models [9] expect more symmetric division in *l* type of environment, it may be expected that cells with more than one aggregates and distribution of these aggregates to both the daughter cells would be more common in *L* and/or *l*. However the total frequency of this phenomenon was so small that this could not be tested. Also at such a low frequency it is unlikely to make a difference to the population.

### Protein aggregates progressively disappear on starvation

**Figure 4:**
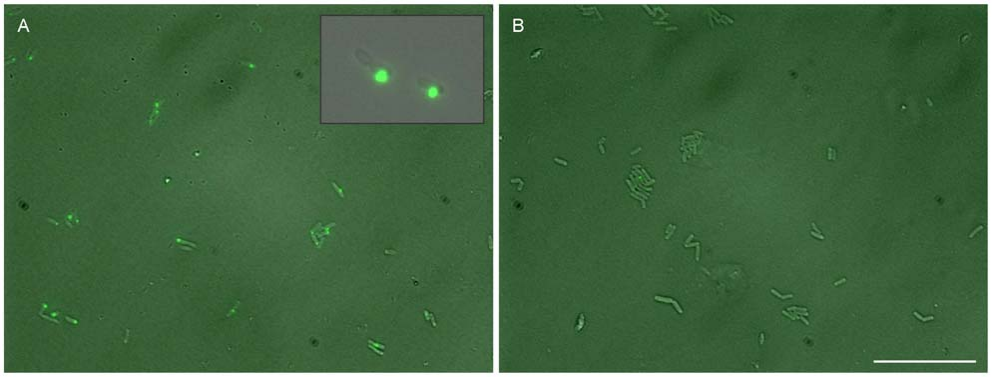
Comparative superimposed DIC and fluorescence images of protein aggregates in cells immediately after entering the stationary phase. (A) and after starvation for 4 days (B). The scale bar represents 20 µ. Inset: some of the larger aggregates did not disappear after prolonged starvation (30 days) but altered the morphology showing diffused fluorescence sometimes even exceeding the cell boundary.

**Figure 5:**
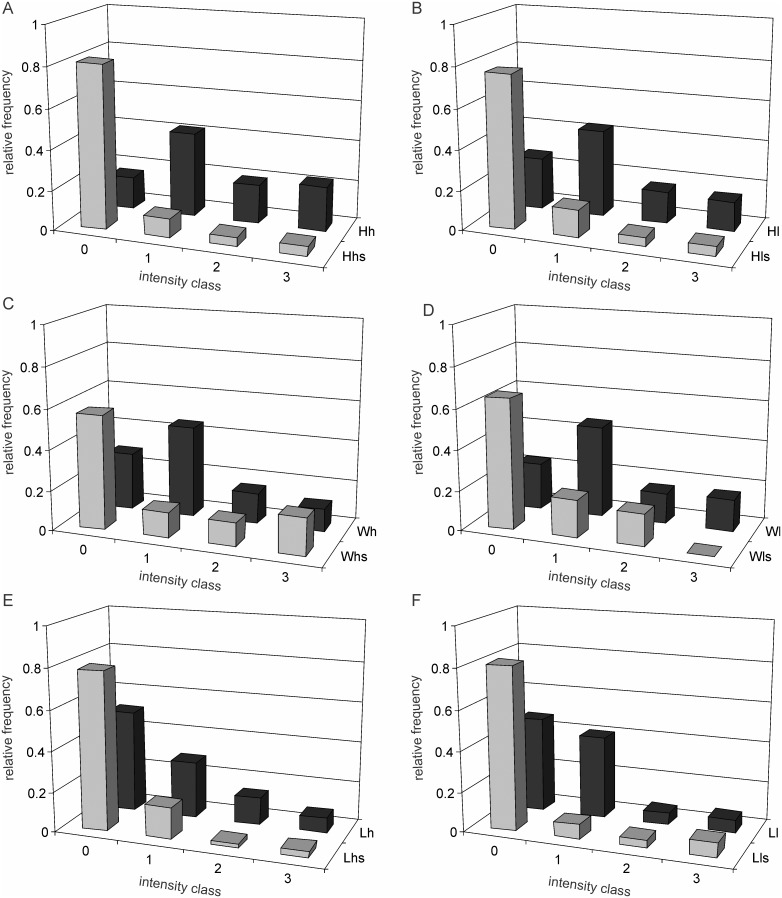
The distribution of intensity classes before and after starvation for 4 days. A–F represents *Hh, Hl, Wh, Wl, Lh* and *Ll* respectively. The suffix *s* stands for starvation. Although the distribution before starvation was different in all selection-concentration combinations, the distribution after starvation is similar. In all lines the aggregate free class increased significantly and class 1 reduced consistently. The trend in class 2 and 3 was less consistent.

**Figure 6:**
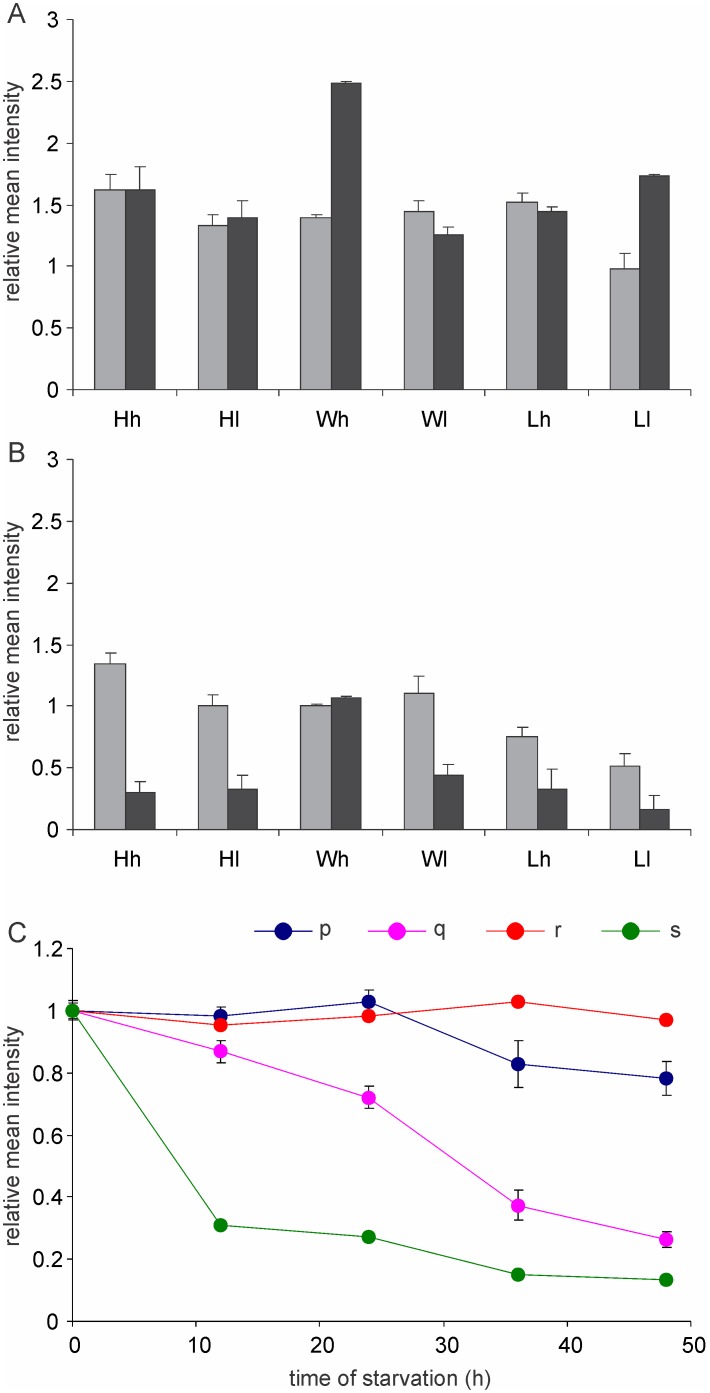
The effect of 4 day’s starvation on visible protein aggregates. (n = 100±20 each for data in Fig. 2A to C). A and B: The mean fluorescence intensities before (grey bars) and after (black bars) starvation for 4 days. Means calculated excluding (A) and including (B) aggregate free cells. All columns are significantly reduced in B but not reduced or significantly increased in A. This suggests that all aggregate classes are not degraded proportionately. Many of the larger ones could be relatively resistant to degradation while the smaller ones degrade fast, thereby increasing the mean excluding aggregate free cells after degradation. *denotes significant difference from Wh. $ denotes significance between corresponding starved and unstarved populations (at p<0.05). C: The time course of decrease in the mean fluorescence intensity with starvation: The intensities are expressed relative to the mean intensity at zero hours. p: represents mean excluding aggregate free cells and q: represents including them in continued stationary phase. r: represents averages excluding aggregate free cells and s: represents including aggregate free cells in distilled water. Error bars show standard error of the mean.

In order to test whether the aggregated proteins are degraded and recycled on facing starvation we compared aggregate distribution in cells at the end of exponential phase with the same culture continued in the stationary phase for 4, 8, 15 and 30 days. By the 4^th^ day itself the proportion of aggregate free cells had increased in all lineages (Fig. 4). In the 8^th^, 15^th^ and 30^th^ day pictures, smaller aggregate classes were not observed, some large aggregates were still seen but with an altered morphology and diffused fluorescence around them, the fluorescence occasionally diffusing out of the cell (Fig. 4 inset). The distribution of intensity classes was substantially altered by 4 day starvation (Fig. 5) in all selection lines. The proportion of no aggregate cells increased and that in intensity class 1 decreased consistently. In *Hh* there was a 5 fold rise in the proportion of aggregate free cells and 80% decline in class 1. On the other hand since *Lh* already had over 50% cells in the aggregate free class there was a 1.54 fold rise and class 1 declined by 46%. There was no consistent trend in classes 2 and 3. Although the trends in classes 2 and 3 are difficult to infer because of smaller frequencies, it is possible that the frequency in these classes does not decline as rapidly as class 1. The mean fluorescence intensity was reduced substantially on starvation when the aggregate free cells were included in the average calculation. However, when these cells were excluded the mean intensity did not decrease significantly or actually increased in some groups (Fig. 6). This indicates that the aggregates were not being degraded proportionately. The smaller aggregates were more likely to be degraded rapidly the larger ones being relatively resistant to degradation. The occasional appearance of large aggregates in 8, 15 and 30 day starved cells with diffusing fluorescence suggests that at least some of the large aggregates may not be degraded at all. This could be either because above a critical size aggregation might become irreversible, or cells containing very large aggregates may be dead. The dynamics of large aggregate degradation, if any, could be qualitatively different than the smaller ones.

**Figure 7:**
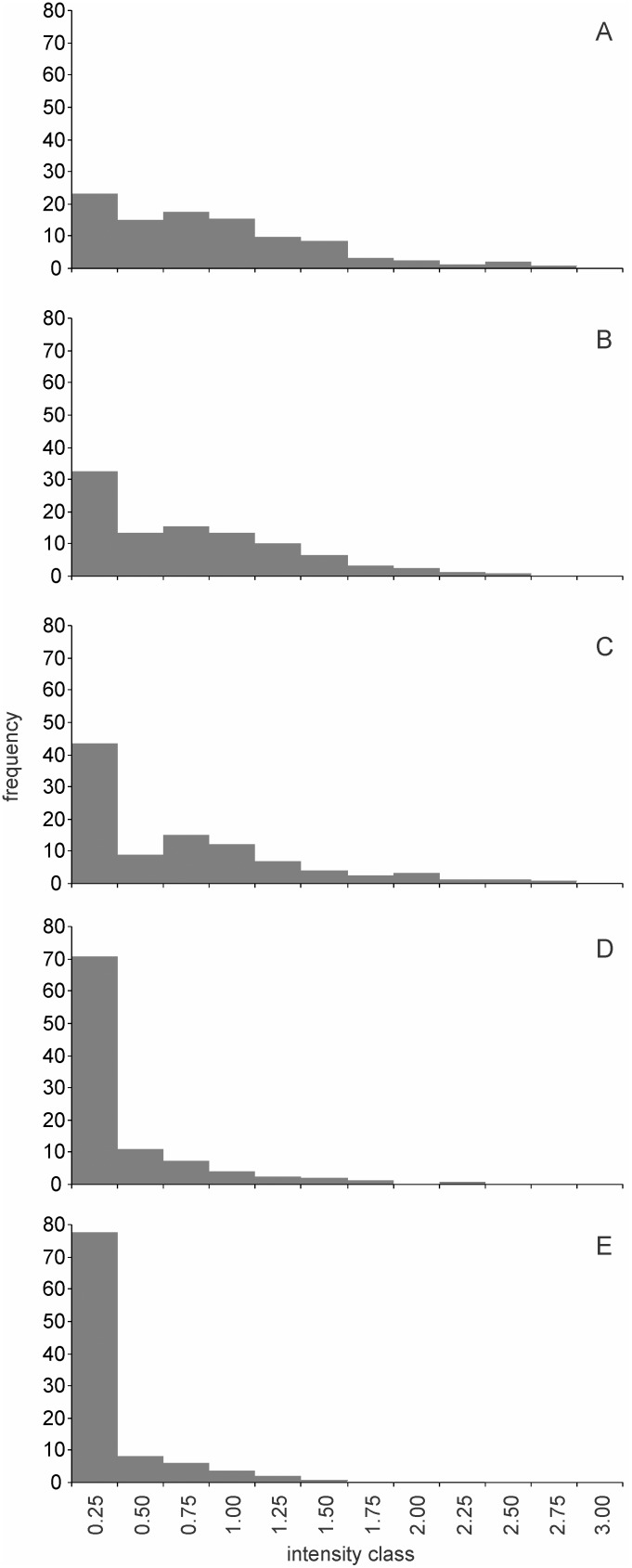
The progressively changing frequency distribution of fluorescence intensities on exposure to starvation. A: at the beginning of stationary phase B to E: 12, 24, 36, and 48 hours of starvation respectively. The intensities are classified at a higher resolution but the scale is matched with that in other figures. Note the transient bimodality created presumably due to faster degradation of smaller aggregate classes. Trends were similar but somewhat slower (not shown here) when cells were followed in the same medium after reaching stationary phase. The transient bimodality was consistently seen across both the conditions.

In order to get a finer time as well size resolution we studied the fate of protein aggregates during stationary phase of *Wh* at every 12 hours using a larger sample size (500 to 800 cells each). The cells were either incubated in the same medium or washed and transferred to distilled water and both were observed under fluorescence microscopy after every 12 hours up to 48 hours. In the continued stationary phase experiment there was no significant reduction in the viable count whereas during and after washing and incubation in distilled water the viable count decreased by approximately one third. The pattern of change in the frequency distribution of fluorescence intensities with time can help us resolve between alternative hypotheses for the reduction in the mean intensities with time. Apart from the hypothesis of interest that the protein aggregates are degraded and recycled during starvation, the other possible explanations for the reduction in mean intensity and increase in the proportion of aggregate free cells are (i) the fluorescent protein has a short half life compared to the duration of the experiment and therefore fluorescence decreases with time (ii) senescent cells with large PAs die and disintegrate thereby reducing the mean fluorescence in the surviving population (iii) some replication continues in the stationary phase but there is little protein aggregation owing to low nutrient availability and therefore the proportion of aggregate free cells increases. The three alternative explanations make differential predictions that are testable by observing the changes in mean intensities and the frequency distribution of intensities with time. (i) GFP and YFP are known to be stable proteins [29] and therefore are unlikely to have disintegrated during the experimental period. We observed some large PAs retaining fluorescence up to 30 days (Fig. 4) indicating that fluorescence could remain substantially longer. Exponential reduction in fluorescence should give rise to a greater reduction in the high intensity classes than in the low intensity classes on a linear scale. This does not match with the observed pattern of decrease in the mean (Fig. 6 C) as well as frequency distributions (Fig. 7). Also we observed a significant decrease in the insoluble protein fraction (IPF) of the cells which reduced from 0.613 at the beginning of starvation to 0.267 at the end of 72 hours supporting the hypothesis that the aggregates actually degraded and half life of fluorescence is not an adequate explanation of the observed decline. (ii) If the reduction in mean is brought about by the death of cells with large PAs the right hand tail of the distribution would be curtailed first and the reduction in the mean excluding zero class should be comparable or faster than the reduction in the mean including zero class. This did not match with the observed pattern (Fig. 7). Also when the cells were incubated in continued stationary phase we did not observe a decrease in the total viable count of cells in early stationary phase which makes this explanation unlikely. (iii) If new cells are born without aggregates, the frequency of zero class would increase but there would be no change in the mean calculated excluding zero class. The mean excluding aggregate free cells did not decrease in some and decreased marginally in other sets in this study which is compatible with this hypothesis. However, in order to explain the observed pattern in the decline of mean and the frequency distribution, the population should have increased between 1.5 to 1.8 fold. This is unlikely for a starving population. Also the total viable count did not increase either in continued stationary phase or in distilled water. In distilled water the viable count decreased whereas in continued stationary phase it did not. However, in spite of this difference the percent reduction in fluorescence in the two was not significantly different. Therefore we do not consider selective growth or death as adequate explanations of the observed decline.

A closer look at the pattern of change in the frequency distribution (Fig. 7) reveals that there appears a transient bimodality during early (12 and 24 hours) starvation. This is compatible with our earlier suggestion that the smaller aggregates disappeared at a faster rate than the rate at which the larger ones reduced their size.

### Correlation between visible protein aggregates, estimates of insoluble protein fraction and RNA content of cells

**Figure 8:**
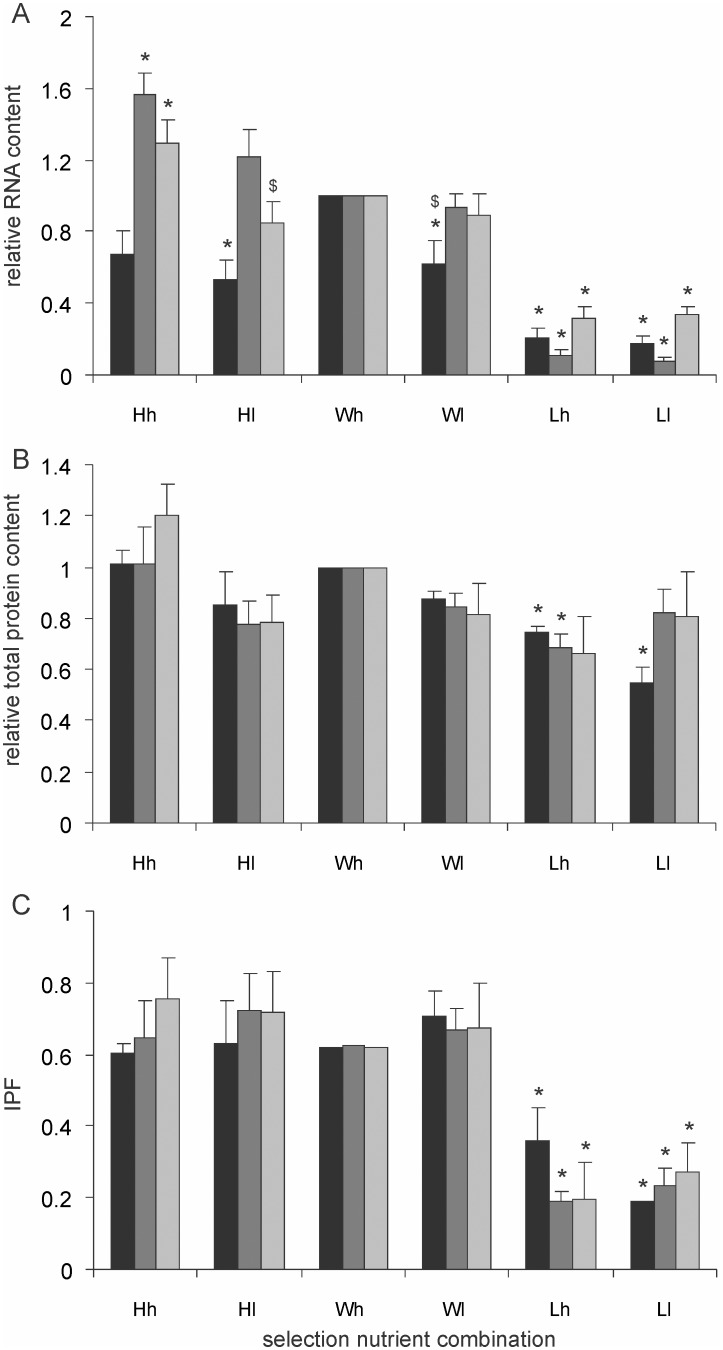
RNA and protein contents of cells. (A) Total RNA (ng/µl) (B) Total protein (µg/ml) and (C) The fraction of insoluble protein (IPF) content of the selection lines under high and low current nutrition. All are expressed relative to Wh. Black columns stand for KL16, dark grey for 2563 and light grey for MGAY. RNA contents differed over an order of magnitude in the *L* selected lines. *denote significant difference from *Wh*. $ denote significant difference between corresponding *h* and *l* conditions.

The standing RNA content (Fig. 8A) of a cell is a direct indication of metabolic activity and therefore we expect the RNA levels to be different under different nutritional conditions and under differential selection. For all the three strains and in each of the three replicates we use the *Wh* as a reference and express the chemistry of all other lineages relative to it. Therefore throughout the results *Wh* has a mean of unity and variance of zero. Results show that the total RNA content of cells was substantially different. The RNA content of *l* was lower than that of *h* in 8 out of the 9 pairs. Selection had larger and significant effects. In all the three strains *L* selected lines had significantly lower RNA content under both *h* and *l* conditions as compared to *W* and *H* lines. The RNA contents were not consistently affected by *H* selection across all the three strains.

**Figure 9:**
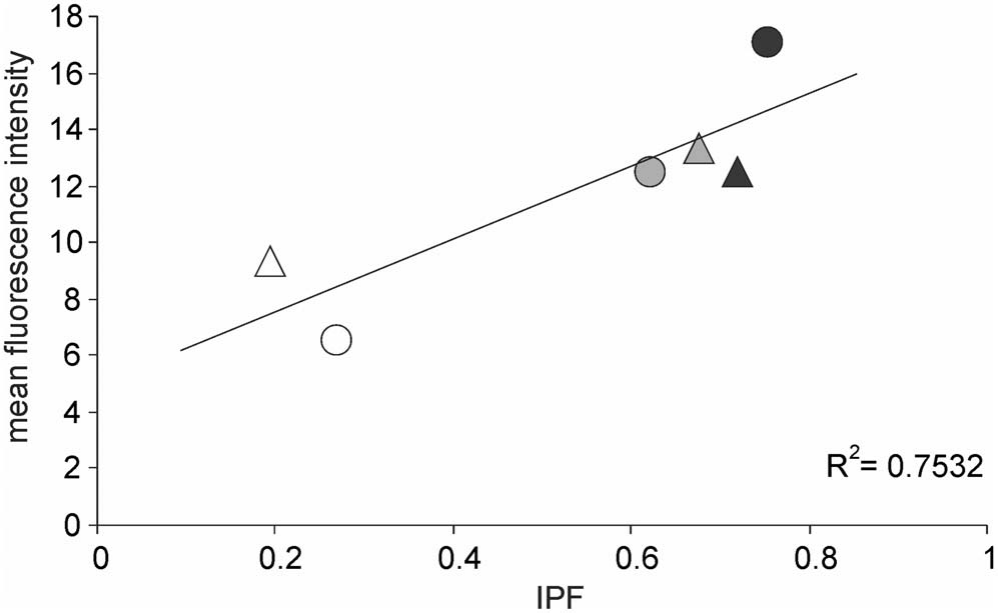
Correlation between mean fluorescence intensity and insoluble protein fraction in MGAY. The circles denote *h* and triangles denote *l* conditions (r = 0.868, n = 6, p<0.05). Open circles and triangles stand for *L* selection, grey for *W* and black for *H*. The correlation validates the use of IPF as a surrogate for protein aggregation in strains without a fluorescent marker.

**Figure 10:**
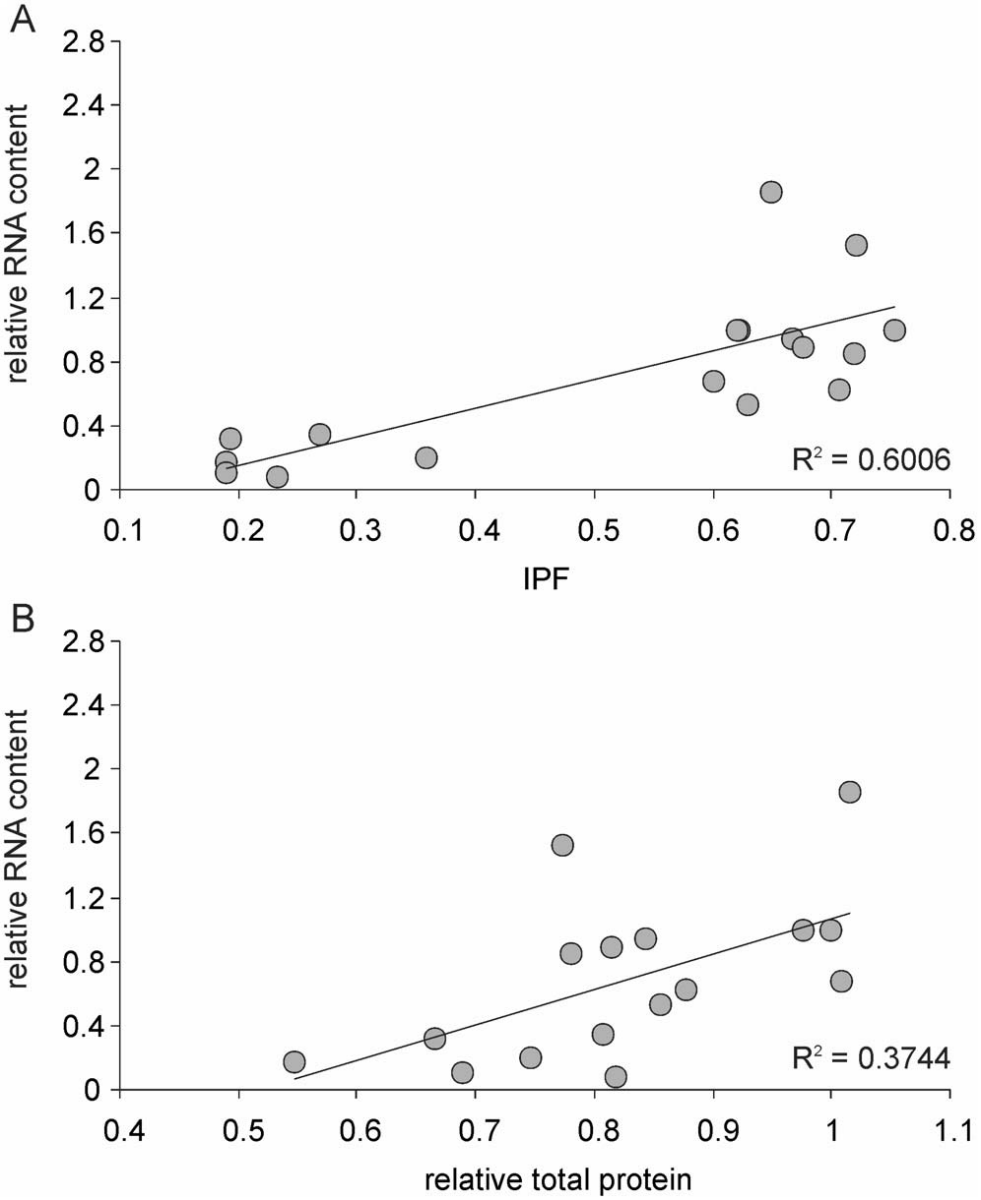
Correlation with total RNA content of insoluble protein fraction (r = 0.7749, n = 18, p<0.05) and the total protein (r = .612, n = 18, p<0.05) in the pooled data over all the strains and their selection lines.

The total protein contents (Fig. 8B) of the cells also followed a similar pattern. In 7 out of the 9 pairs *l* conditions showed lower cell protein content than *h* conditions. Selection played a greater role, particularly *L* having a substantial reduction effect in all the three strains. More dramatic were the differences in soluble versus insoluble fractions in cell proteins (Fig. 8C). There was no consistent pattern across *l* and *h* conditions but *L* selection altered the ratio of insoluble to soluble proteins by almost an order of magnitude. Protein aggregates contribute to the insoluble fraction and therefore the two findings that *L* selection reduces visible protein aggregates and also reduces the fraction of insoluble proteins are mutually supportive. In MGAY there was good correlation between the mean fluorescence intensity of protein aggregates and IPF (Fig. 9). Therefore we can justifiably use IPF as a surrogate for protein aggregation in the other selection lines too. The total protein as well as insoluble protein fraction correlated significantly positively with total RNA content in the pooled data over all the strains and their selection lines (Fig. 10).

The relation between nutritional environment and functional cell division time asymmetry has been shown earlier [11]. If protein aggregates are responsible for cell division asymmetry, we expect our data to correlate with earlier results. Correlations of the insoluble fraction with cell division time asymmetry, frequency of senescent cells, growth rates and growth yields of selection lines derived from KL16 and 2563 (obtained from Lele *et al.* 2011) [11] were not significant parametrically. However when separately ranked for the two strains under the two growth conditions, ranked correlations across the three selection regimes were significantly positive between IPF and asymmetry index (r = 0.625, n = 12, p<0.005) and significantly negative between IPF and growth rate (r = −0.625, n = 12, p<0.05). The proportion of senescent cells in the three selection regimes pooled over *h* and *l* and ranked separately for the two strains correlated positively with IPF (r = 0.875, n = 6, p<0.05). This indicates that the two strains differ substantially in the pattern of quantitative response to *h* and *l* but the direction of response to selection is similar and therefore after correcting for the strain difference and current nutritional environment, protein aggregation is significantly positively correlated with cell division asymmetry and the frequency of senescent cells and negatively with growth rate in a non-parametric correlation. The correlation with growth yield nevertheless remained insignificant even after the correction. It is observed from the combined results of Lele *et al* [11] and this work that growth yield is affected significantly by *h* and *l* conditions whereas protein aggregation and cell division symmetry by *H* and *L* selection.

## Discussion

### Protein aggregation has an evolvable component

We showed previously that caloric restriction in short as well as long term reduces asymmetry in cell division time in *E. coli* and thereby the frequency of cells showing the signs of aging [11]. This study primarily demonstrates a relationship between caloric restriction and protein aggregation in the species supporting the hypothesis that the effect of caloric restriction on cell division symmetry and aging might be mediated through altered proteostasis. In all the three strains studied, in the lines selected under low nutrient environments there was less protein aggregation as compared to those selected in nutrient rich environments. The effect of nutrients on protein aggregation can potentially be a direct effect of nutrient concentration on the metabolic dynamics or it could be an evolved strategy of the cell to handle proteins differently under different nutrient conditions. The long term selection experiment gives support to the latter although the former is not eliminated. The quantitative effects of selection on protein aggregation suggest that there is some evolvable genetic control over protein aggregation although chemically and thermodynamically protein misfolding and aggregation may be an inevitable process. The possible cellular control over protein aggregation appears to operate more effectively at an early stage before visible protein aggregates appear since the different selection lines differ substantially in the proportion of visibly aggregate free cells but within cells showing aggregates, the frequency distribution of intensity or the spatial distribution along the cell length is only marginally different (Fig. 3). This has important implications for the inevitability versus evolved strategy debate. There appears to be substantial strategic control on protein aggregation, but the asymmetric distribution of the aggregates to the daughter cells is invariant and not responsive to nutrient based selection.

Lele *et al.* [11] demonstrated selection for symmetry in different strains of *E. coli* independently subject to the same selection regime. Protein aggregation could not be demonstrated in these strains by using fluorescence, but we showed here that the insoluble protein fraction showed response to selection in the same direction. Further in MGAY in which both IPF and visible aggregates can be monitored, there was a good correlation between fluorescence intensity of visible aggregates and IPF. Therefore IPF can be used as a reliable surrogate for protein aggregation. This allows us to test the generality of the results across different strains. In all the three strains selection in low nutrient environment reduced the tendency to aggregate proteins and selection in high nutrient concentration increased it independent of the effects of standing nutrient concentration. This illustrates the generality and reproducibility of the phenomenon. Unlike some of the earlier studies that used heat or some other stress to induce protein aggregation [24], [30], [31] we observed the phenomenon here with no such external inducer of protein denaturation. Also we made a onetime cross sectional observation without repeatedly exposing the organisms to fluorescence. This is important in the light of the objection raised on the earlier studies that the observed protein aggregation and aging may be a response to the stress rather than being a natural phenomenon [23].

### Selection on protein handling?

Since protein aggregation is related to aging in *E. coli* [4], [6], [32], [33], the demonstration that caloric restriction reduces protein aggregates has relevance to aging. It appears that the phenomenon of caloric restriction increasing the lifespan begins with bacteria along the hierarchy of life and here the mechanism is likely to be much simpler as compared to multicellular organisms. In bacteria this phenomenon can perhaps be more easily interpreted simultaneously at proximate and ultimate levels. Although many gaps in data still remain, it appears that when nutrient availability is good, the cells may adopt a use and throw strategy in handling proteins because it allows rapid growth [9] albeit with reduced energy efficiency. On the other hand in nutrient poor environments, energy efficiency could be more important for survival and therefore recycling of proteins is more adaptive than dumping them in the form of aggregates. It is easy to understand why such a strategy would be adaptive in a resource poor environment.

### Is there any advantage of protein aggregates?

Our findings point to another dimension of protein aggregation that aggregation is not the end point. The aggregated proteins can be and seem to be reutilized when the cells face starvation. Within four days of starvation the proportion of aggregate free cells increases significantly in all selection lines. But interestingly within the cells that retain aggregates, the mean aggregate intensity is only marginally decreased in some, not significantly decreased, or in fact increased in some cases. If all aggregates degraded with some rate, both the number of aggregate free cells and mean intensity of aggregates should have decreased proportionately. The more sluggish change in the later indicates that the larger aggregates do not degrade as efficiently. Some of them remain undegraded till 30 days. The rate of reutilization of aggregates is likely to be negatively associated with the size of protein aggregates. The smaller aggregates disappear faster on facing starvation and the larger ones appear to linger on disproportionately. This could be simply an effect of decrease in surface to volume ratio of the aggregates or aggregates above a threshold may interfere with some metabolic functions of the cell directly or indirectly making the cell non-viable. This has implications for the biology of aging. If protein aggregation is a mechanism of aging, the reutilization of aggregates points to the possibility of reversibility of aging. Not only that, the aggregated proteins may facilitate survival during starvation. These possibilities need to be tapped in near future.

### Implications for the evolution of aging

Many issues in the evolution of aging are debated over a long time and bacterial aging is likely to provide some new insights into it [34]. The demonstration of aging in *E. coli* was taken as a convincing evidence for the universality and therefore inevitability of aging for the living world. Although many of the mechanisms leading to aging including oxidative damages, protein aggregation, telomere shortening, glycosylation etc are inevitable, the patterns of aging are not universal across species [35]. The probability of death and reproductive potential does not necessarily decrease with age in all life forms. The reutilization of protein aggregates raises a possibility that a senescent bacterial cell with a protein aggregate may have better chances of surviving starvation although this has not yet been directly demonstrated.

The reutilization of protein aggregates has a few other possible implications too. *E. coli* is not known to store fat as energy reserve which most multicellular animals do. In humans the tendency to store fat has been popularly known as “thrift” and is said to have evolved as a result of repeated exposure to starvation [36], [37], [38]. There are three close parallels between fat storage in higher animals and protein aggregates in *E. coli*. In both the cases high energy intake increases the storage of fat/proteins, under starvation both can be reutilized and excessive adiposity as well as protein aggregates appear to negatively affect at least some of the normal metabolic processes. This suggests that *E. coli* can be a good model to study the evolution of thrift. Although this was not the objective of the present study, it makes certain suggestions in this context. Continued exposure to low nutrient availability appears to increase the metabolic efficiency, compatible with the thrift hypothesis, but it does not result into excessive storage/aggregation. Instead growth at low nutrients increases growth yield and the efficiency of reutilization rather than the efficiency of storage. Thus there are similarities as well as differences with the prevalent concept of thrift in humans. This warrants further evolutionary experiments specifically designed to probe the evolution of thrift in a bacterial model.

The correlation of protein aggregates with metabolic rate is consistent with the Watve *et al.* [9] hypothesis prediction that asymmetric segregation should increase the net metabolic rate of the population although the cells with aggregates would slow down. Although we did not directly measure metabolic rate, the order of magnitude difference in the standing RNA content probably reflects the increased rate of transcription and thereby metabolism in general. It is important to realize that although we showed that the growth rate is negatively correlated with protein aggregation, this correlation is done after correcting for the main growth rate determining factor that is the currently available nutrient concentration. Without this correction the correlation was positive but non-significant. More surprising is the order of magnitude difference in the total DNA content which most probably reflects the standing chromosomal copy number and simultaneous replication forks [39]. This difference has many implications for the mutation dynamics, mutation tolerance, neutral space and evolvability of the organism. This is an intricate mathematical problem which we do not want to explore here. It is interesting to note that there is a substantial change in the overall cell chemistry indicating some fundamental alterations in metabolic regulation as a result of selection. The cells evolve to become more dilute in terms of protein and nucleic acid concentrations when they evolve in dilute environments.

There are likely to be other direct or indirect benefits of aging like processes in bacteria. Growth arrest accompanying protein aggregation is suggested to give temporary resistance to antibiotics [40]. For accruing this benefit too, the aging process needs to be reversible in bacteria. If such cells have a greater probability of surviving antibiosis or prokaryotic predation [41] and they can reutilize the protein aggregates, the population has a better chance of rejuvenating. Even if a senescent cell with large protein aggregate may have lost its ability to rejuvenate, the stored proteins may be available as a nutrient source to neighboring cells after the death and degeneration of the senescent cell. Thus there can be multiple possible advantages of senescence in bacteria which makes a good case for senescence being an adaptively evolved phenomenon rather than being an inevitable fate. Also since some of the advantages such as better chances of surviving starvation are possible individual advantages and others such as better growth rate are population advantages, both programmed and non-programmed aging are likely to be operative in bacteria. Alternatively the programmed versus non-programmed aging debate may in fact be irrelevant to bacteria [34].
